# CTDSPL2 promotes the progression of non-small lung cancer through PI3K/AKT signaling via JAK1

**DOI:** 10.1038/s41420-024-02162-5

**Published:** 2024-08-29

**Authors:** Muzi Li, La Chen, Fangfang Yu, Huijuan Mei, Xingxing Ma, Keshuo Ding, Yanan Yang, Ziye Rong

**Affiliations:** 1 Department of Immunology, School of Basic Medical Sciences, Anhui Medical University, Hefei, Anhui China; 2 Department of Pathology, School of Basic Medical Sciences, Anhui Medical University, Hefei, Anhui China; 3 Department of Pathology, the First Affiliated Hospital of Anhui Medical University, Hefei, Anhui China

**Keywords:** Non-small-cell lung cancer, Tumour biomarkers

## Abstract

Carboxy-terminal domain small phosphatase like 2 (CTDSPL2), one of the haloacid dehalogenase phosphatases, is associated with several diseases including cancer. However, the role of CTDSPL2 and its regulatory mechanism in lung cancer remain unclear. Here, we aimed to explore the clinical implications, biological functions, and molecular mechanisms of CTDSPL2 in non-small cell lung cancer (NSCLC). CTDSPL2 was identified as a novel target of the tumor suppressor miR-193a-3p. CTDSPL2 expression was significantly elevated in NSCLC tissues. Database analysis showed that CTDSPL2 expression was negatively correlated with patient survival. Depletion of CTDSPL2 inhibited the proliferation, migration, and invasion of NSCLC cells, as well as tumor growth and metastasis in mouse models. Additionally, silencing of CTDSPL2 enhanced CD4^+^ T cell infiltration into tumors. Moreover, CTDSPL2 interacted with JAK1 and positively regulated JAK1 expression. Subsequent experiments indicated that CTDSPL2 activated the PI3K/AKT signaling pathway through the upregulation of JAK1, thereby promoting the progression of NSCLC. In conclusion, CTDSPL2 may play an oncogenic role in NSCLC progression by activating PI3K/AKT signaling via JAK1. These findings may provide a potential target for the diagnosis and treatment of NSCLC.

## Introduction

Lung cancer is one of the most prevalent malignant tumors with high incidence and mortality worldwide, particularly NSCLC, which is the most common pathological type, representing approximately 80%–85% [1]. Accumulating evidences have shown that alternations of various genes and signaling pathways play a crucial role in the pathogenesis and progression of NSCLC [2–4]. In the past decade, important advances have been made in the treatment of NSCLC with the development of targeted therapies and immunotherapeutic agents [5]. The clinical application of mutant epidermal growth factor receptor targeted tyrosine kinase inhibitors and programmed cell death-ligand 1 (PD-L1) immune checkpoint inhibitors has greatly benefited patients [6, 7]. Nevertheless, the survival rates of NSCLC patients remain poor [8]. Therefore, exploring novel molecular targets and their underlying mechanisms is imperative for enhancing the diagnosis and treatment of NSCLC.

CTDSPL2, alternatively referred to as SCP4 or HSPC129, is a serine/threonine phosphatase [9]. CTDSPL2 is primarily localized in the nucleus, particularly in transcriptionally inactive heterochromatin regions. However, it can also be detected in the cytoplasm [10]. CTDSPL2, although part of a family of proteins with potential for drug targeting, has received limited research attention. CTDSPL2 is involved in regulating glucose metabolism. CTDSPL2-mediated dephosphorylation of FoxO1/3a is critical for promoting hepatic gluconeogenesis and muscle proteolysis [11]. Reports have suggested that CTDSPL2 is implicated in tumor development, with phosphatase activity being a contributing factor. CTDSPL2 overexpression in chick embryo fibroblasts promotes cell migration and survival [12]. CTDSPL2 dephosphorylates and stabilizes Snail, thereby enhancing TGFβ-induced epithelial-mesenchymal transition (EMT) [13]. In pancreatic cancer cells, CTDSPL2 knockdown upregulates p21 and p27 expression, resulting in the suppression of cell proliferation, migration and invasion by inducing mitotic defects [14]. In acute myeloid leukemia (AML), CTDSPL2 dephosphorylates the kinases STK35 and PDIK1L, supporting the proliferation of AML cancer cells and affecting the expression of genes involved in amino acid biosynthesis and transport [15]. However, until now, the biological functions and molecular mechanisms underlying the role of CTDSPL2 in NSCLC progression are completely undefined.

By RNA sequencing and TCGA analysis, we found that CTDSPL2 is a novel target gene of the tumor suppressor miR-193a-3p, which has the potential to function as an oncogenic molecule that promotes NSCLC progression. This study demonstrated for the first time that CTDSPL2 expression levels are elevated in NSCLC tissues. Knockdown of CTDSPL2 impeded the growth and metastasis of NSCLC cells both in vitro and in vivo. We further explored the downstream signaling pathway and found that CTDSPL2 promoted the progression of NSCLC by activating the PI3K/AKT pathway through its interaction with and upregulation of JAK1. Furthermore, CTDSPL2 depletion promoted CD4^+^ T cell infiltration within tumors. The findings are expected to establish a theoretical basis for the development of targeted therapies for NSCLC.

## Results

### CTDSPL2, a potential novel target of the tumor suppressor miR-193a-3p, is upregulated in NSCLC and associated with poor patient survival

The inhibitory effect of miR-193a-3p on tumor progression has been reported in many reports [16–19]. However, the specific downstream target acts as an oncogenic gene in NSCLC is yet to be identified. In this experiment, miR-193a-3p-overexpressing stable cells were generated in H1299 cells, and miR-193a-3p mimics were transiently transfected into A549 cells (Supplementary Fig. 1A, B). Our results confirmed that the overexpression of miR-193a-3p inhibited the proliferation, migration, and invasion of NSCLC cells (Supplementary Fig. 1C–H).

**Fig. 1:**
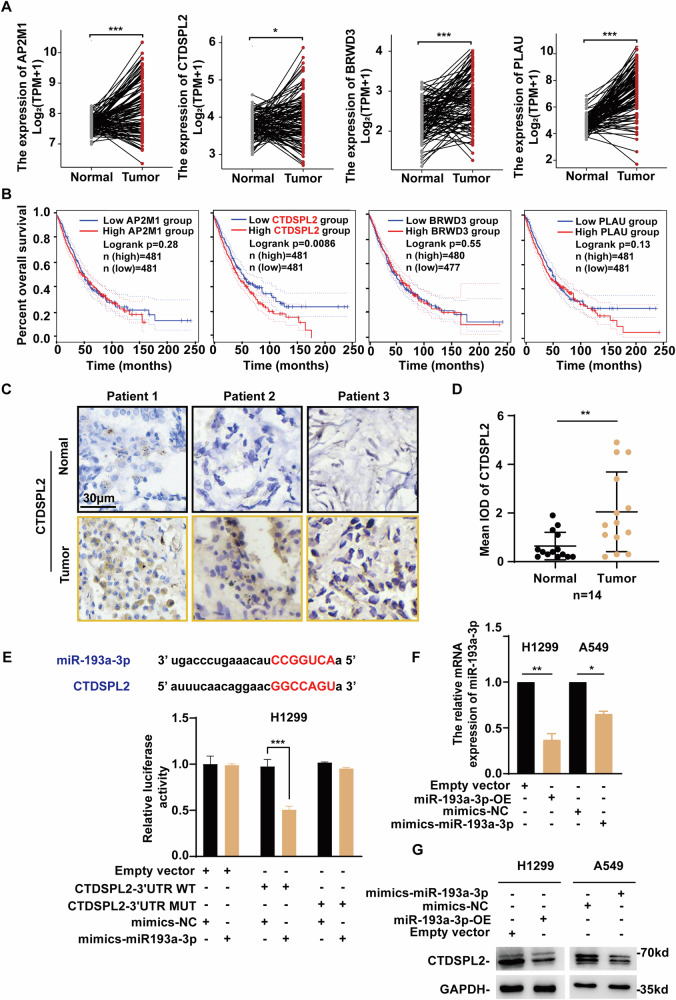
CTDSPL2, a direct target of the tumor suppressor miR-193a-3p, is highly expressed in NSCLC and associated with poor patient survival. **A** Differential expression of the AP2M1, CTDSPL2, BRWD3, and PLAU between NSCLC tumor tissues and matched normal tissues from TCGA database. **B** Effects of the 4 genes on patient overall survival were analyzed by GEPIA2. **C** Representative images of IHC staining of CTDSPL2 (brown) on noncancerous and cancerous lung tissue sections from NSCLC patients. **D** Quantification analysis of CTDSPL2 expression in tissue samples in (**C**). Mean ± SD, **p < 0.01. **E** The binding site of miR-193a-3p to CTDSPL2 predicted and validated using TargetScan and dual luciferase reporter assay, respectively. **F**, **G** qRT-PCR and western blot analysis of CTDSPL2 expression in cells overexpressed miR-193a-3p. Mean ± SEM, *p < 0.05, **p < 0.01, ***p < 0.001.

To search for the potential targets implicated in promoting NSCLC progression, we first predicted the target genes of miR-193a-3p using four databases: TargetScan, miRDB, PicTar, and miRanda. A Venn diagram was generated with an online tool (http://bioinformatics.psb.ugent.be/webtools/Venn/) and a total of 56 shared target genes were obtained (Supplementary Fig. 2A). We subsequently conducted transcriptome sequencing of H1299 cells stably expressing miR-193a-3p or miR-NC. Using a combination of database prediction and sequencing results, we analyzed the changes in the expression of these 56 common genes following miR-193a-3p overexpression. Among them, 12 were upregulated and 10 were downregulated, as shown in the heatmap (Supplementary Fig. 2B). Subsequently, TCGA database was used to compare the expression levels of the 10 downregulated genes between tumor and normal tissues in NSCLC patients. The findings indicated that the mRNA levels of AP2M1, CTDSPL2, BRWD3 and PLAU were significantly elevated in cancerous tissues (Fig. 1A and Supplementary Fig. 2C). Then overall survival (OS) analysis was performed using GEPIA2 (http://gepia2.cancer-pku.cn/#index). Among the 4 candidates, only higher expression of CTDSPL2 was correlated with a poorer survival rate in patients with NSCLC (Fig. 1B). The association between CTDSPL2 expression and survival outcome was confirmed using Kaplan-Meier plotter (https://kmplot.com/analysis/) (Supplementary Fig. 2D). In addition, CTDSPL2 expression at different stages was investigated in LUAD and LUSC by GEPIA2. The results revealed that CTDSPL2 was differentially expressed in LUAD [F value = 2.78, Pr (>F) = 0.0409] but not in LUSC (Supplementary Fig. 2E). To further evaluate CTDSPL2 protein expression in NSCLC tissues, we performed immunohistochemical staining of paraffin-embedded tissue samples from 14 patients with NSCLC. The protein expression level of CTDSPL2 was higher in tumor tissues than that in non-tumor tissues (Fig. 1C, D). This finding was consistent with the results obtained from TCGA data analysis. Therefore, CTDSPL2 is highly expressed in NSCLC and may have potential oncogenic functions.

### CTDSPL2 is directly targeted by miR-193a-3p in NSCLC cells

TargetScan prediction revealed a putative binding site between miR-193a-3p and the 3’-UTR of CTDSPL2 (Fig. 1E). To validate the direct targeting of the CTDSPL2 3’-UTR by miR-193a-3p, a dual luciferase reporter assay was performed. As shown in Fig. 1E, the wild-type or mutant 3’-UTR of CTDSPL2 was cloned into the luciferase reporter plasmid. Overexpression of miR-193a-3p significantly reduced luciferase activity of the wild-type CTDSPL2 3’-UTR, but not the mutant 3’-UTR, suggesting that CTDSPL2 was a direct target of miR-193a-3p (Fig. 1E). Next, we evaluated the effect of miR-193a-3p overexpression on CTDSPL2 expression in NSCLC cells (H1299 and A549). Our data revealed that ectopic miR-193a-3p expression inhibited CTDSPL2 expression at both the mRNA and protein levels (Fig. 1F, G). Together, CTDSPL2 is negatively regulated by miR-193a-3p through direct targeting, indicating that CTDSPL2 elevation in NSCLC could be attributed to decreased miR-193a-3p expression.

### CTDSPL2 promotes malignant progression of NSCLC cells

**Fig. 2:**
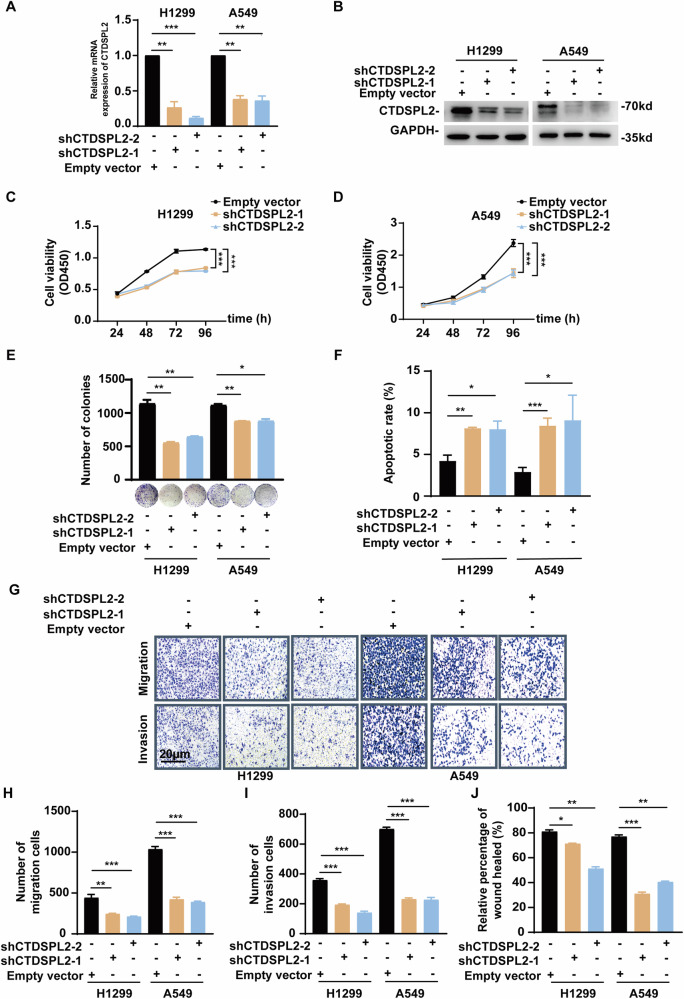
CTDSPL2 promotes malignant biological functions in lung cancer cells. qRT-PCR (**A**) and western blot (**B**) analysis validating the effect of CTDSPL2 knockdown. **C**, **D** CCK-8 assay was used to evaluate the proliferation of NSCLC cells depleted with CTDSPL2. **E** Representative images and quantification of colony formation assay. **F** Quantification of cell apoptosis by flow cytometry. Representative images (**G**) and quantitative statistical analysis of the effects of CTDSPL2 knockdown on NSCLC cell migration (**H**) and invasion (**I**) using transwell assays. **J** Quantification of wound healing assay to evaluate cell migration. Mean ± SEM, *p < 0.05, **p < 0.01, ***p < 0.001.

To investigate the function of CTDSPL2 in NSCLC, lentiviral shRNA transduction was used to stably knockdown CTDSPL2 in H1299 and A549 cells (Fig. 2A, B). The CCK8 assay demonstrated a significant decrease in NSCLC cell proliferation upon silencing of CTDSPL2 (Fig. 2C, D), which was further confirmed by colony formation assay (Fig. 2E). To explore the mechanism by which CTDSPL2 influences cell proliferation, we examined the cell cycle distribution and cell apoptosis using flow cytometry. CTDSPL2 depletion resulted in G1 phase arrest and increased apoptosis in both the cell lines (Supplementary Fig. 3 and Fig. 2F). Additionally, transwell assays were conducted to determine the effect of CTDSPL2 on migration and invasion. Inhibition of CTDSPL2 significantly attenuated the migratory and invasive abilities of NSCLC cells (Fig. 2G–I). Wound-healing assay was also used to assess the migratory ability of the cells. NSCLC cells lacking CTDSPL2 exhibited reduced wound closure rates (Fig. 2J and Supplementary Fig. 4). Collectively, these findings demonstrate that CTDSPL2 plays an oncogenic role by inhibiting apoptosis and facilitating the proliferation, cell cycle progression, migration, and invasion of NSCLC cells.

### CTDSPL2 facilitates tumor growth and metastasis in mouse models, while inhibiting the infiltration of CD4^+^ T cells into tumor tissues

**Fig. 3:**
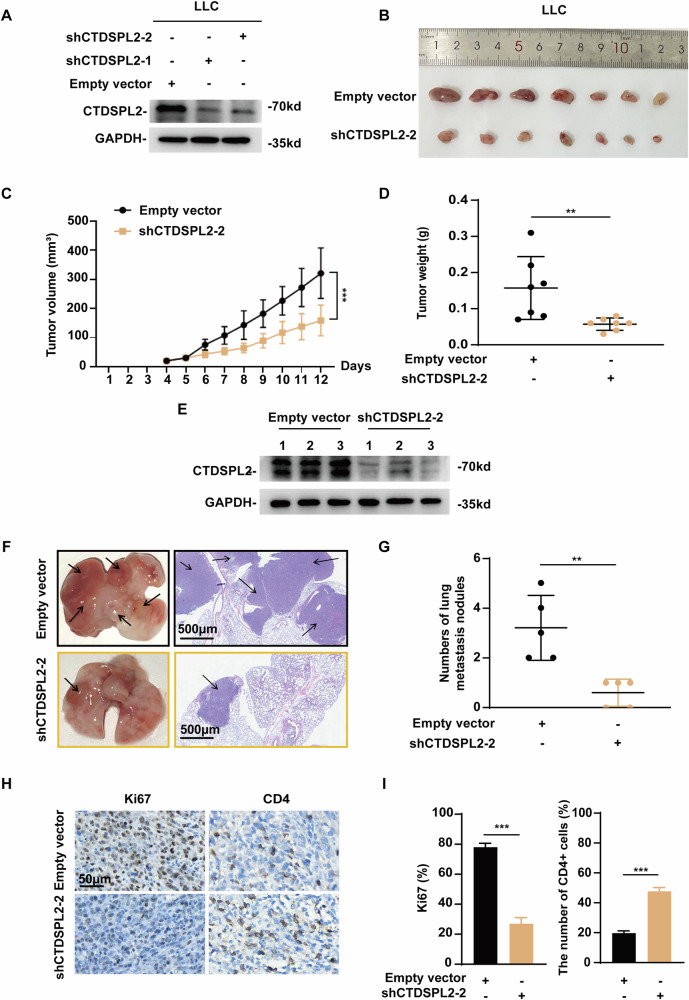
CTDSPL2 depletion impedes tumor growth and metastasis in cell-derived mouse tumor models and promotes tumor infiltration by CD4^+^ T cells. **A** Validation of western blot results for CTDSPL2 knockdown in LLC cell lines. **B** Tumors from C57/BL6 mice subcutaneously injected with LLC-derived cells (7 mice each group). **C** Tumor growth curve showing the effect of CTDSPL2 expression on tumor volume. Tumor volume was calculated as 0.5 × length × width^2^. **D** Statistical plot illustrating the effect of CTDSPL2 expression on tumor weight. **E** Western blot analysis of CTDSPL2 expression in tissue lysates extracted from three random tumors in each group. **F** Left panel: Representative images of lungs from C57/BL6 mice after injection of LLC-derived cells via the tail vein (5 mice each group). Right panel: H&E staining of corresponding lung sections. Black arrows indicate the metastatic nodules. **G** Statistical analysis of the number of lung metastasis nodules. **H**, **I** Expression of Ki67 and CD4 in subcutaneous tumors obtained in (**B**). Mean ± SEM, **p < 0.01, ***p < 0.001.

In our animal experiments, a murine lung cancer cell line, LLC, was used to stably deplete CTDSPL2 (Fig. 3A). CTDSPL2 knockdown or control cells were subcutaneously injected into C57/BL6 mice and tumor formation was monitored. Tumors derived from the CTDSPL2 knockdown group exhibited reduced growth rates, smaller size, and lighter weight than the control group (Fig. 3B–D). Furthermore, IHC staining revealed that expression of the proliferation marker Ki-67 was reduced upon CTDSPL2 silencing (Fig. 3H). The expression of CTDSPL2 protein in tumor tissues was examined by western blot analysis. The results confirmed a reduction in CTDSPL2 expression in tumor tissues from the shCTDSPL2 group (Fig. 3E). The above LLC-derived cells were also inoculated into the tail vein of C57/BL6 mice. The CTDSPL2 knockdown group exhibited reduced metastatic foci in lung tissues (Fig. 3F, G). Overall, these data suggested that CTDSPL2 significantly enhanced NSCLC tumor growth and metastasis in vivo.

Using the tumor tissues from immune-competent C57/BL6 mice subcutaneously injected with LLC-derived cells, we performed IHC staining to evaluate the influence of CTDSPL2 on T cell recruitment. The shCTDSPL2 group exhibited increased infiltration of CD4^+^ T cells into tumor tissues (Fig. 3H, I). Hence, our findings demonstrated for the first time that CTDSPL2 suppresses CD4^+^ T cell infiltration in tumors. Further investigation is necessary to evaluate the effects of CTDSPL2 on different CD4^+^ T cell subsets.

### CTDSPL2 promotes lung cancer progression by activating the PI3K/AKT pathway via the regulation of JAK1 expression

**Fig. 4:**
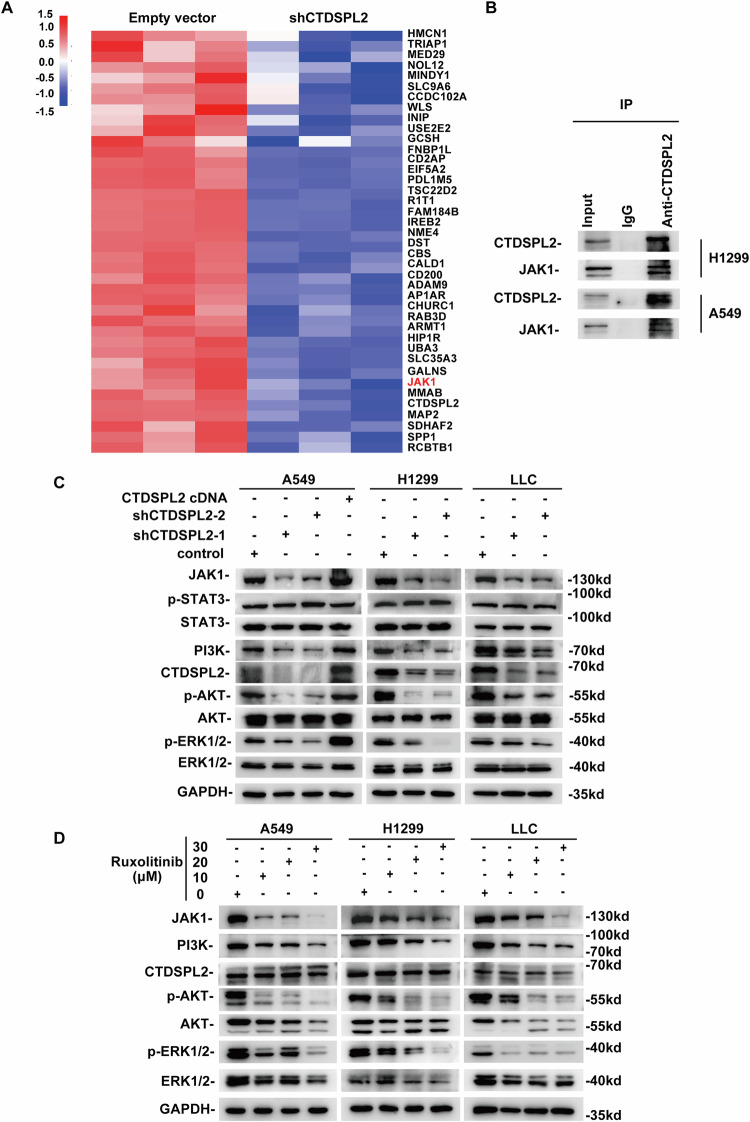
CTDSPL2 interacts with JAK1 and upregulates its expression to activate PI3K/AKT signaling. **A** TMT quantitative mass spectrometry heatmap depicting the differential expression of top 40 downregulated proteins in the CTDSPL2 knockdown group. **B** Immunoprecipitation experiment results validating the binding ability of CTDSPL2 to JAK1 in different lung cancer cells. **C** Western blotting analysis of indicated proteins in CTDSPL2 knockdown group, CTDSPL2 overexpression group, and control group. **D** Western blot results verifying the inhibition of the PI3K/AKT signaling pathway in lung cancer cells after ruxolitinib treatment.

In an attempt to elucidate the underlying molecular mechanisms by which CTDSPL2 promotes the malignant progression of NSCLC, tandem mass tagging (TMT) quantitative proteomics analysis was applied to compare H1299-shCTDSPL2 cells with control cells. Heatmap visualization revealed the top 40 downregulated proteins, among which JAK1 attracted our interest (Fig. 4A). A previous report has implicated the interaction between CTDSPL2 and JAK1 in HeLa cells by mass spectrometry [20]. This interaction was also validated by immunoprecipitation of endogenous CTDSPL2 (Fig. 4B). In NSCLC, activation of the JAK1/STAT3 pathway is considered to be crucial for tumor progression [21–23]. Additionally, JAK also mediates other signaling pathways involved in tumor progression, such as PI3K pathway [24, 25]. Accordingly, the effect of CTDSPL2 on the expression of key components of the JAK1/STAT3 and PI3K/AKT signaling pathways was investigated by western blot assay. The results revealed that depletion of CTDSPL2 reduced the expression of JAK1, PI3K, p-AKT(Ser473), and p-ERK1/2(Thr202/Tyr204) in H1299, A549, and LLC cells (Fig. 4C). However, altering CTDSPL2 expression did not affect the levels of STAT3, p-STAT3(Tyr705), AKT, or ERK1/2 proteins (Fig. 4C). Meanwhile, overexpression of CTDSPL2 showed the opposite results (Fig. 4C). To assess the effect of JAK on PI3K/AKT signaling, we administered different doses of ruxolitinib, a selective JAK1/2 inhibitor, to three lung cancer cell lines. As expected, JAK inhibition resulted in a decrease in the expression of PI3K, p-AKT, and p-ERK1/2 (Fig. 4D). In contrast, JAK inhibition had minimal effects on CTDSPL2 expression, suggesting that CTDSPL2 acted as a regulator of JAK1. To further confirm the effect of JAK1, cells were transfected with siRNA oligos against JAK1. Similar with ruxolitinib treatment, specific knockdwon of JAK1 reduced PI3K and p-AKT levels, but not CTDSPL2 and total AKT levels (Supplementary Fig. 5).

**Fig. 5:**
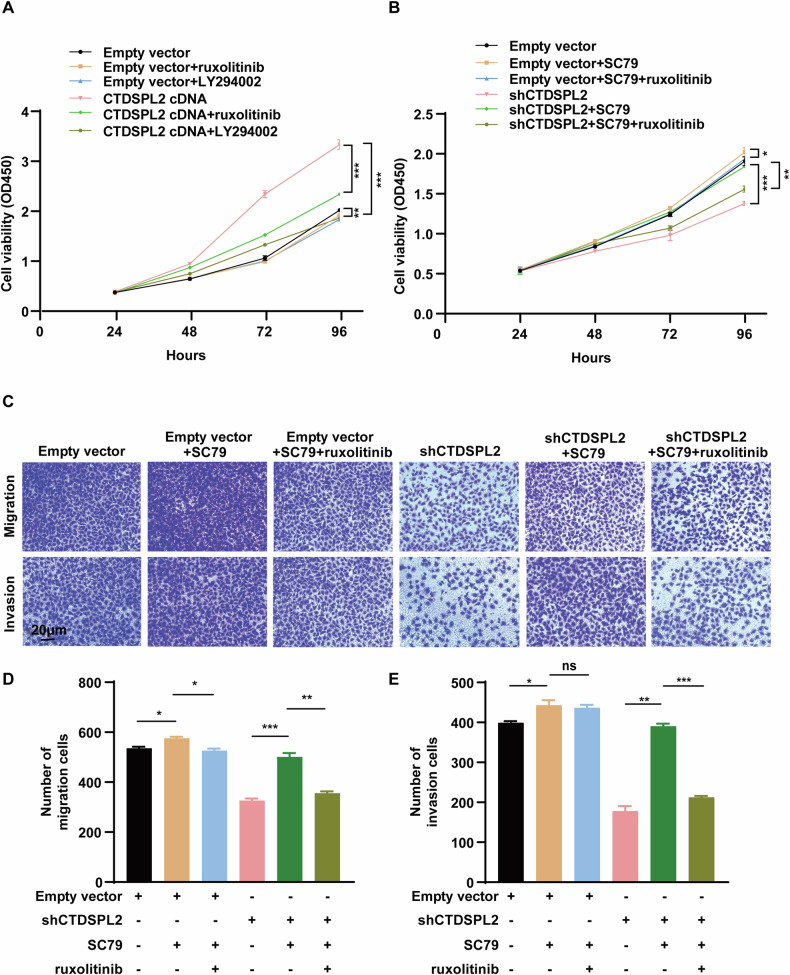
CTDSPL2 promotes lung cancer progression through the JAK1/PI3K/AKT axis. **A** CCK-8 assay evaluating the effects of ruxolitinib or LY294002 treatment on the proliferation of control and CTDSPL2-overexpressed A549 cells. **B** CCK-8 assay evaluating the effects of SC79 with or without ruxolitinib on the proliferation of control and CTDSPL2-depleted A549 cells. Representative images (**C**) and quantitative statistical analysis of the effects of the indicated drug treatments on cell migration (**D**) and invasion (**E**) using transwell assays. Mean ± SEM, *p < 0.05, **p < 0.01, ***p < 0.001.

**Fig. 6:**
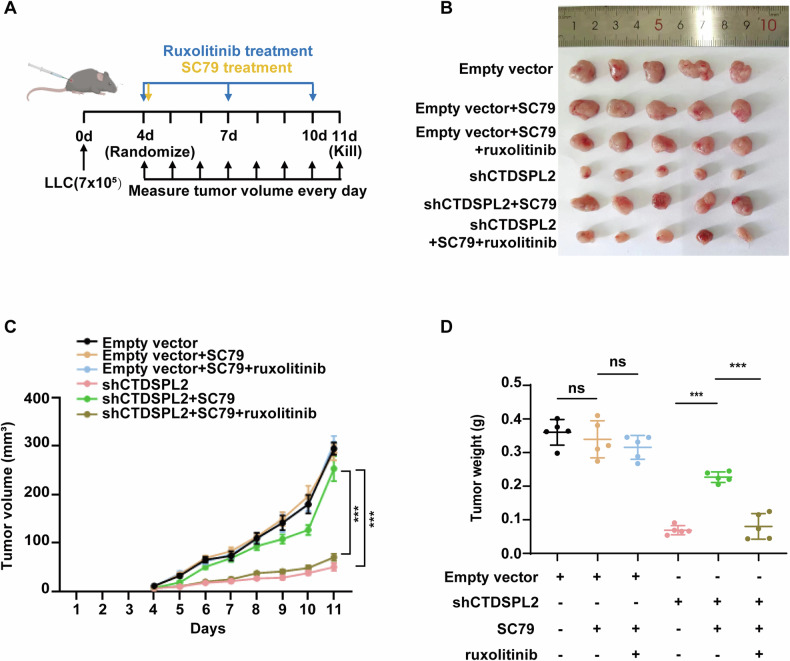
CTDSPL2 improves tumor growth through the JAK1/PI3K/AKT axis in vivo. **A** Schema of drug treatment experiment. 4 days after subcutaneous inoculation of LLC-derived cells, mice were randomized into different treatment groups receiving SC79 alone, SC79 with ruxolitinib, or PBS through intraperitoneal injection. **B** Tumors from subcutaneous tumor-bearing mice treated with different drugs (5 mice each group). **C** Tumor growth curve of tumors in (**B**). **D** Statistical plot illustrating the weight of tumors in (**B**). Mean ± SEM, ***p < 0.001.

**Fig. 7:**
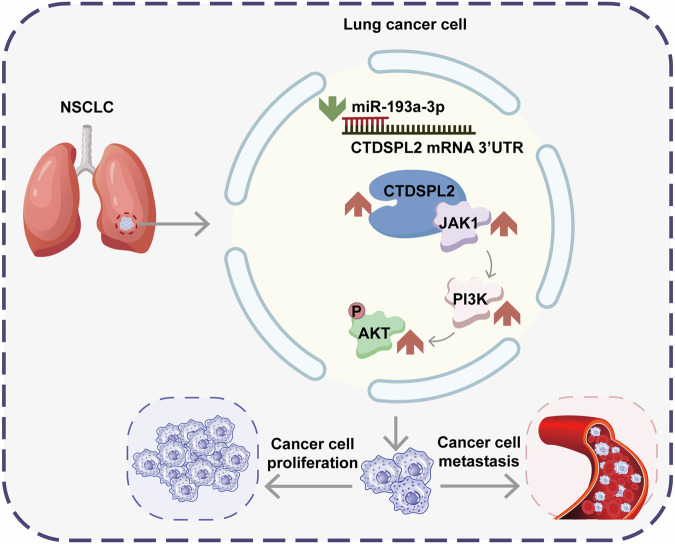
Schematic illustration of the role of CTDSPL2 in NSCLC progression through the JAK1/PI3K/AKT signaling axis. Decreased miR-193a-3p expression in NSCLC tissues leads to upregulation of CTDSPL2, which activates the PI3K/AKT signaling by interacts with JAK1 and upregulates its expression. This activation promotes the malignant biological behavior of NSCLC cells, including cell proliferation and metastasis.

To investigate the potential of CTDSPL2 to promote NSCLC cell proliferation via JAK1/PI3K/AKT signaling, we administered ruxolitinib (20 μM) or LY294002 (20 μM), a PI3K inhibitor, to A549 cells stably expressing CTDSPL2. CCK8 assay demonstrated that CTDSPL2 overexpression-induced lung cancer cell proliferation was reversed by inhibiting JAK or PI3K (Fig. 5A). Control cells treated with ruxolitinib or LY294002 also showed a slight decrease in cell proliferation (Fig. 5A). Then, SC79, an AKT activator, was used to enhance AKT phosphorylation. An increase in cell proliferation was observed in A549 cells treated with SC79 (20 μM). Furthermore, SC79 mitigated the inhibitory effect of CTDSPL2 knockdown on cell proliferation (Fig. 5B). Interestingly, this rescue effect was partially eliminated when ruxolitinib (20 μM) was co-administered with SC79 (Fig. 5B). Similarly, SC79 treatment promoted cell migration and invasion. Decreased migration and invasion caused by CTDSPL2 knockdown were also rescued by SC79 treatment, while co-administration of ruxolitinib with SC79 abolished this rescue effect (Fig. 5C–E). Next, we used subcutaneous tumor-bearing mice to further confirm the involvement of JAK1/PI3K/AKT axis in CTDSPL2-induced tumor growth. 4 days after LLC-derived cell injection, the mice were treated with drug as indicated in Fig. 6A. SC79 treatment partially rescued attenuated tumor growth caused by CTDSPL2 knockdown and such effect was reversed by ruxolitinib combined with SC79 treatment (Fig. 6B–D). In summary, these findings suggest that CTDSPL2 potentially promotes NSCLC progression by activating the PI3K/AKT pathway through its interaction with and upregulation of JAK1 (Fig. 7).

## Discussion

NSCLC, the most common lung cancer type, is still the leading cause of cancer death worldwide. Despite recent advances in the management of NSCLC, treatment of these patients continues to pose a clinical challenge that needs to be resolved [8, 26]. Therefore, identifying novel biomarkers and elucidating the underlying mechanisms of NSCLC is imperative.

MiR-193a-3p expression is typically reduced in NSCLC tissues compared with adjacent noncancerous tissues, indicating its role as a tumor suppressor [18]. Accumulating evidences have demonstrated that miR-193a-3p inhibits cell proliferation and invasion in NSCLC via different target genes [17, 27]. RNA-seq and database analyses were performed to screen novel miR-193a-3p targets in NSCLC to discover new oncogenic molecules.

This study investigated the functional role and underlying molecular mechanisms of CTDSPL2 in NSCLC progression after identifying it as a novel target of the tumor suppressor miR-193a-3p. CTDSPL2 has been reported to play a critical role in glucose metabolism, cell migration, and cell survival [11, 12]. Recent developments have emerged regarding the association between CTDSPL2 and tumors [14, 15]. However, the precise role of CTDSPL2 in NSCLC has not been clarified.

Based on the database analysis, we demonstrated for the first time that CTDSPL2 is highly expressed in NSCLC tissues, and higher expression of CTDSPL2 is associated with lower survival rates. Our experimental findings with clinical specimens confirmed that CTDSPL2 is elevated in NSCLC tissues compared with normal lung tissues. In vitro functional assays revealed that CTDSPL2 deletion suppressed malignant behaviors, including cell proliferation, migration, and invasion, in NSCLC cells. In a syngeneic mouse model, we validated that CTDSPL2 knockdown impeded tumor growth and metastasis in vivo. Our findings indicate, for the first time, that CTDSPL2 functions as an oncogene in NSCLC. Thus, CTDSPL2 may serve as a promising therapeutic target for NSCLC.

Several reports have implicated the involvement of phosphatase activity of CTDSPL2 in tumor development. CTDSPL2-mediated dephosphorylation of Snail inhibits its degradation, consequently enhancing TGFβ-induced EMT [13]. In AML, CTDSPL2 dephosphorylates the kinases STK35 and PDIK1L, facilitating their interaction, influencing the expression of genes related to amino acid biosynthesis and transport, and promoting the proliferation of AML cells [15]. However, whether CTDSPL2 promotes NSCLC progression through the dephosphorylation of specific substrates remains unclear. Further investigations are warranted to explore the significance of CTDSPL2 phosphatase activity and its downstream substrates in NSCLC progression.

Mechanistic studies have demonstrated a crucial role of JAK1 as a downstream effector of CTDSPL2. Subsequent investigations have indicated that CTDSPL2 has the potential to influence the activation of the PI3K/AKT pathway via JAK1, which is important for CTDSPL2-induced NSCLC progression. JAK1 has been implicated in the progression of several human cancers, including lung cancer [28–31]. However, the specific regulatory mechanism by which CTDSPL2 mediates JAK1 requires further research. As shown in Supplementary Fig. 6, the transcription of JAK1 was unaffected by CTDSPL2. Hence, we hypothesized that CTDSPL2 could regulate JAK1 protein levels by inhibiting its ubiquitination and subsequent degradation. An additional question pertains to the potential involvement of the phosphatase activity of CTDSPL2 during this process. Epigenetic studies and mass spectrometry will assist in elucidating the intricate downstream mechanisms associated with the function of CTDSPL2 in NSCLC.

Furthermore, we present novel evidence that CTDSPL2 inhibits the infiltration of CD4^+^ T cells into tumors. CTDSPL2 may potentially affect T cell infiltration in tumors via JAK1, as previous research has demonstrated the influence of JAK1 on the tumor microenvironment [32–35]. CTDSPL2 may influence CD4^+^ T cell tumor infiltration through additional pathways. Furthermore, discerning alterations in distinct CD4^+^ T cell subtypes following CTDSPL2 depletion is imperative. An intriguing question that remains to be answered is whether infiltration of other immune cells is correlated with CTDSPL2. Single-cell sequencing and fluorescence-activated cell sorting analyses in a syngeneic mouse model hold promise for addressing these questions.

In conclusion, our study elucidated the role of CTDSPL2, a novel downstream target gene of miR-193a-3p, in NSCLC progression. CTDSPL2 can promote the malignant progression of NSCLC, thereby enhancing tumor growth and metastasis. Furthermore, CTDSPL2 knockdown restored CD4^+^ T cell infiltration. Mechanistically, we speculated that CTDSPL2 could promote NSCLC progression by activating the PI3K/AKT signaling pathway via JAK1. Our findings contribute to the understanding of CTDSPL2 function in NSCLC and enhance our knowledge of the underlying molecular mechanisms, which have significant implications in the development of targeted therapies for NSCLC.

## Materials and methods

### Clinical specimens

Paraffin-embedded cancerous and noncancerous lung tissue samples from 14 patients with NSCLC were obtained from the Department of Pathology of the First Affiliated Hospital of Anhui Medical University. The clinical information of the samples is listed in Supplementary Table 1. This study was approved by the Biomedical Ethics Committee of Anhui Medical University (No. 82230043).

### Cell culture and chemicals

A549, H1299 and LLC cells were acquired from the Cell Bank of the Chinese Academy of Sciences (Shanghai, China). All cells were grown in Dulbecco’s modified Eagle’s medium (Wisent, Nanjing, China) supplemented with 10% fetal bovine serum and 1% penicillin-streptomycin. All cells were authenticated by STR profiling and tested for mycoplasma contamination. Ruxolitinib and SC79 were purchased from AbMole (USA) and LY294002 was from Med Chem Express (Shanghai, China).

### Cell transfection

Lentiviral vectors for miR-193a-3p overexpression (Norbio, Shanghai, China), CTDSPL2 knockdown (Tsingke, Beijing, China), CTDSPL2 overexpression (Miaoling Bio, Shanghai, China), and corresponding empty lentiviral vectors were used to produce lentivirus in 293 T cells with lipo2000 (Thermo Fisher, Shanghai, China). After infecting NSCLC cells with the desired lentivirus, puromycin (Biosharp, Hefei, China) was added to screen the stable transduced cells. miR-193a-3p mimics (Han Bio, Shanghai, China), siJAK1 oligos (General Biol, Anhui, China), and corresponding negative control (NC) were transfected into cells with lipo2000 according to the manufacturer’s protocols. The shRNA and siRNA sequences used in this study are listed in Supplementary Table 2.

### Western blot

Protein samples from cells or tissues were prepared by adding RIPA buffer containing PMSF and protein phosphatase inhibitor (Beyotime, Shanghai, China). Then, the proteins were separated by SDS-PAGE and blotted with appropriate antibodies. Primary antibodies used are as follows: GAPDH, CTDSPL2, JAK1 (Proteintech Group, Wuhan, China), STAT3, p-STAT3 (Cell Signaling Technology, Shanghai, China), PI3K, AKT, p-AKT, ERK1/2, and p-ERK1/2 (Abmart, Shanghai, China).

### Real-time quantitative PCR (qRT-PCR)

Total RNA was extracted using the Sparkjade® kit (Shandong, China) and reverse transcription was performed using 5×HiScript®II qRT SuperMix (Yeasen, Shanghai, China). qRT-PCR was conducted using Power SYBR Green PCR Master Mix (Yeasen). GADPH or U6 was used as the internal reference control. Relative expression levels of the target genes were calculated by the 2^-ΔΔCt^ method. Sequences of the primers are listed in Supplementary Table 3.

### Dual-luciferase reporter assay

CTDSPL2 WT and MUT 3’-UTR luciferase reporter plasmids were constructed by Genechem (Shanghai, China). H1299 cells were seeded in a 48-well plate and transfected with plasmids and Renilla. miR-193a-3p mimics or NC mimics were separately co-transfected with the aforementioned luciferase reporter plasmids. After 48 h, luciferase activity was detected using TransGen Biotech kit (Beijing, China).

### Immunohistochemistry (IHC)

Immunohistochemistry was performed on 4 μm pathological paraffin sections using a DAB Peroxidase (HRP) Substrate Kit (Vector Labs, CA, USA). Antibodies used are as follows: CTDSPL2 (Proteintech Group), Ki-67 and CD4 (Cell Signaling Technology). The CTDSPL2 protein expression was quantified as mean integrated optical density (mean IOD) using the ImageJ software.

### Cell counting kit-8 (CCK8) and colony formation assays

For the CCK8 assay, cells were seeded in a 96-well plate at a density of 2 × 10^3^/well and cultured for 4 days. 10 μl of CCK-8 reagent (Biogene Medical Technology, Anhui, China) was added to each well at 24, 48, 72, and 96 h, and the absorbance value at 480 nm was measured after 2 h.

For the colony formation assay, cells were seeded in 6-cm cell culture dishes at a density of 1 × 10^3^/dish. After culturing for two weeks, cells were fixed with methanol and stained with crystal violet. Colonies were photographed, and the numbers were counted using ImageJ.

### Transwell and wound healing assays

Transwell chambers (Corning, NY, USA) coated with or without BD Matrigel were used to determine cell invasion or migration abilities. 5 × 10^4^ cells were resuspended with 200 μl of serum-free medium and added to the upper chamber. Cells successfully invaded or migrated to the submembrane surface were fixed and stained with crystal violet. The cells were then photographed using an inverted phase-contrast microscope (Olympus, Tokyo, Japan). Cell numbers were counted and quantified using the ImageJ software.

For the wound healing assay, cells were seeded in a 6-well plate at a density of 2 × 10^5^/well. Once the cells reached full confluence, a wound was made by scratching cells with a 200 µl pipette tip. Wound closure was observed and photographed at 0 and 48 h under an inverted phase-contrast microscope.

### Flow cytometry for cell cycle and apoptosis analysis

Changes in cell cycle and apoptosis ratios were evaluated using a Cell Cycle Flow Assay Kit (Beyotime) and an Apoptosis Flow Assay Kit for APC/7-AAD Double-Stained Cells (Bestbio, Changsha, China), respectively. Data were collected by BD FACScelesta3 (NJ, USA).

### Immunoprecipitation assay

The cell lysates were prepared and mixed with CTDSPL2 antibodies overnight at 4 °C. Rabbit IgG antibodies (Proteintech Group) were used as a negative control. Protein A/G beads (Santa Cruz Biotechnology, Shanghai, China) were added to the mixture and rotate overnight at 4 °C. The beads were then washed, and the bound proteins were eluted and analyzed by western blotting.

### Animal studies

Female C57BL/6 mice (Gempharmatech, Jiangsu, China) aged four weeks were obtained and acclimated for one week before experiments. For the subcutaneous tumor model, 5 × 10^5^ LLC-derived cells were injected subcutaneously into the anterior abdomen of mice. Tumor size was recorded daily with a vernier caliper 4 days after injection. The mice were executed after 12 days, and the tumors were removed, weighed, photographed and fixed. For the metastatic tumor model, the mice were injected with 2 × 10^5^ LLC-derived cells via the tail vein and euthanized after one month. Lung tissues were removed, photographed and fixed for further research. For in vivo drug treatment, subcutaneous tumor-bearing mice were randomized into different treatment groups 4 days after injection of LLC-derived cells. Then SC79 (10 mg/kg) alone or SC79 with ruxolitinib (30 mg/kg, every 3 days) was injected intraperitoneally, while PBS was injected as a control. The mice were executed on day 11, and tumors were analyzed as above. All animal experimental procedures were approved by the Animal Research Ethics Committee of Anhui Medical University of China (No. 20231909).

### RNA sequencing and TMT quantitative proteomics

RNA sequencing was performed and analyzed by Gene Denovo Biotechnology Co. Ltd (Guangzhou, China). H1299 cells overexpressed miR-193a-3p and control cells were used to extract total RNA, with three replicates per group. TMT quantitative proteomics was performed and analyzed by Lumingbio (Shanghai, China). H1299 cells stably knockdown CTDSPL2 and control cells were used to prepare protein samples, with three replicates per group.

### Statistical analysis

Data were analyzed using GraphPad Prism 8.0.2 (263) software. The t-test and two-way ANOVA were used for statistical analysis based on specific objectives and data types. *P < 0.05 was considered statistically significant. The data were collected from at least two independent experiments.

### Supplementary information

SUPPLEMENTAL MATERIAL

SUPPLEMENTAL MATERIAL-original WB

## Data Availability

The data that support the findings of this study are included in the paper. Additional related data are available from the corresponding author upon reasonable request.
