# Supplementary material for: CTDSPL2 promotes the progression of non-small lung cancer through PI3K/AKT signaling via JAK1
Source: Cell Death Discov. 2024 Aug 29;10:389. doi: 10.1038/s41420-024-02162-5 (PMC11362329; doi:10.1038/s41420-024-02162-5)
Supplement: Supplementary file 1 — SUPPLEMENTAL MATERIAL [file 41420_2024_2162_MOESM1_ESM.pdf]

Supplementary figures

Supplementary Fig. 1 Effect of miR-193a-3p overexpression on the cell proliferation, migration and invasion in H1299 and A549 cells. A, B qRT-PCR analysis confirming the overexpression of miR-193a-3p in cells. **C** CCK-8 assay showing the effect of miR-193a-3p overexpression on cell proliferation. **D-F** Transwell assays showing the effect of miR-193a-3p overexpression on cell migration and invasion. **G, H** Wound healing assay demonstrating the effect of miR-193a-3p overexpression on cell migration. Mean ± SEM, *p < 0.05, **p < 0.01, ***p < 0.001.

Supplementary Fig. 2 Identification of novel targets of miR-193a-3p. A Venn diagram showing 56 shared miR-193a-3p target genes predicted by 4 online databases. **B** Heatmap showing differential expression of the 56 genes obtained from (A) in H1299 cells stably overexpressing miR-193a-3p based on RNA sequencing results. Among these, 10 were downregulated. **C** TCGA database analysis of the 10 downregulated genes in normal and NSCLC tissues. **D** Kaplan–Meier plot of overall survival with different expression levels of CTDSPL2 in NSCLC patients using the KM database. **E** CTDSPL2 expression at different stages in LUAD and LUSC analyzed by GEPIA2.

Supplementary Fig. 3 Effect of CTDSPL2 on cell cycle progression and apoptosis induction. A Representative images of cell cycle analysis by flow cytometry. **B, C** Quantification of cell cycle analysis. **D** Representative images of cell apoptosis detected by

flow cytometry. Mean ± SEM, *$p < 0.05$, **$p < 0.01$.

Supplementary Fig. 4 Representative images of cell migration evaluating by wound healing assay.

Supplementary Fig. 5 Western blotting analysis of indicated proteins in control and JAK1 knockdown cells. A The efficiency of JAK1 depletion was detected by western blot and siJAK1 showed no effect on CTDSPL2. **B** Effect of JAK1 on PI3K/AKT pathway-related proteins.

Supplementary Fig. 6 qRT-PCR analysis detecting the effect of CTDSPL2 knockdown on mRNA expression of JAK1. Mean ± SEM, *$p < 0.05$.

Supplementary Figure 1

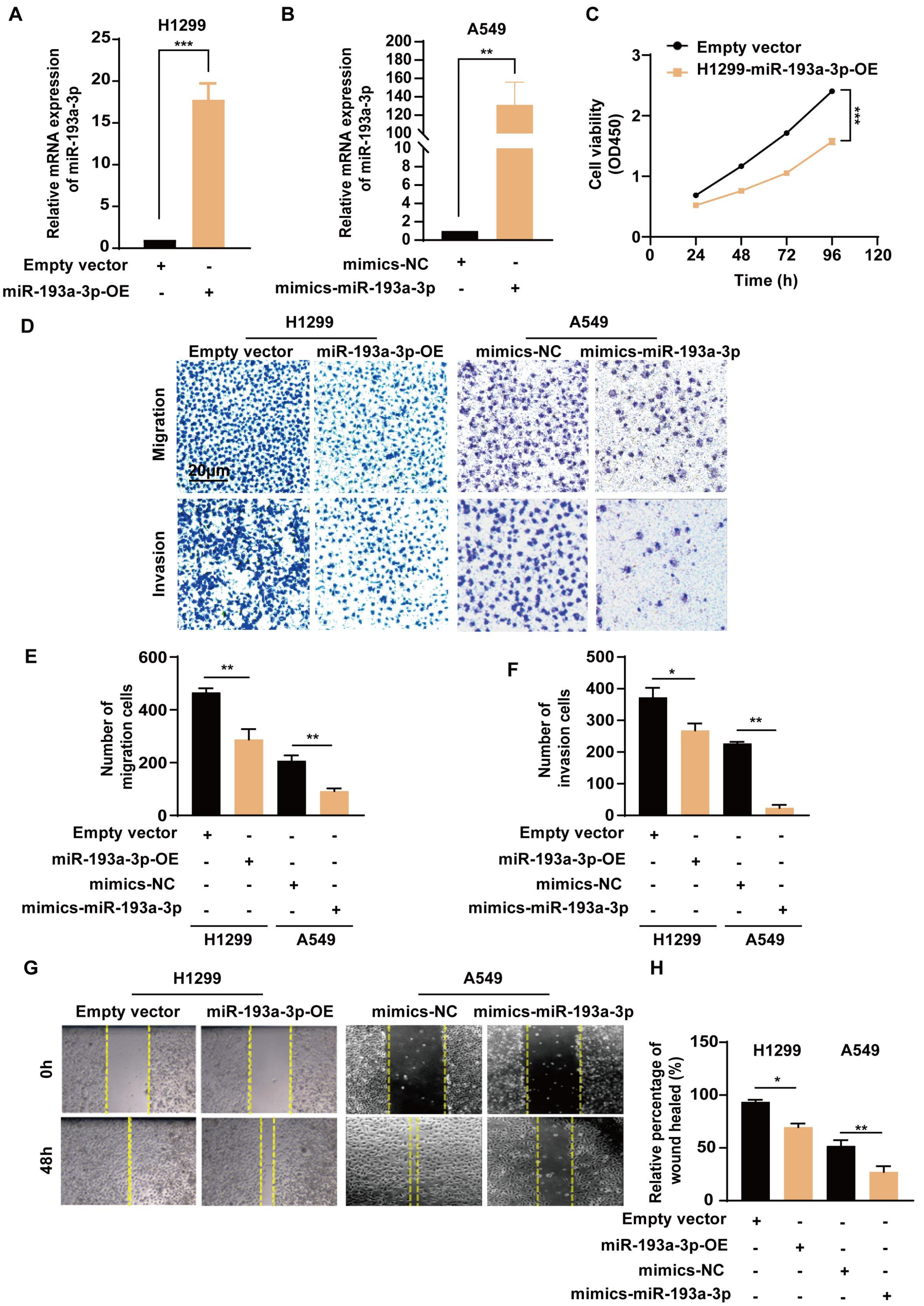

Supplementary Figure 2

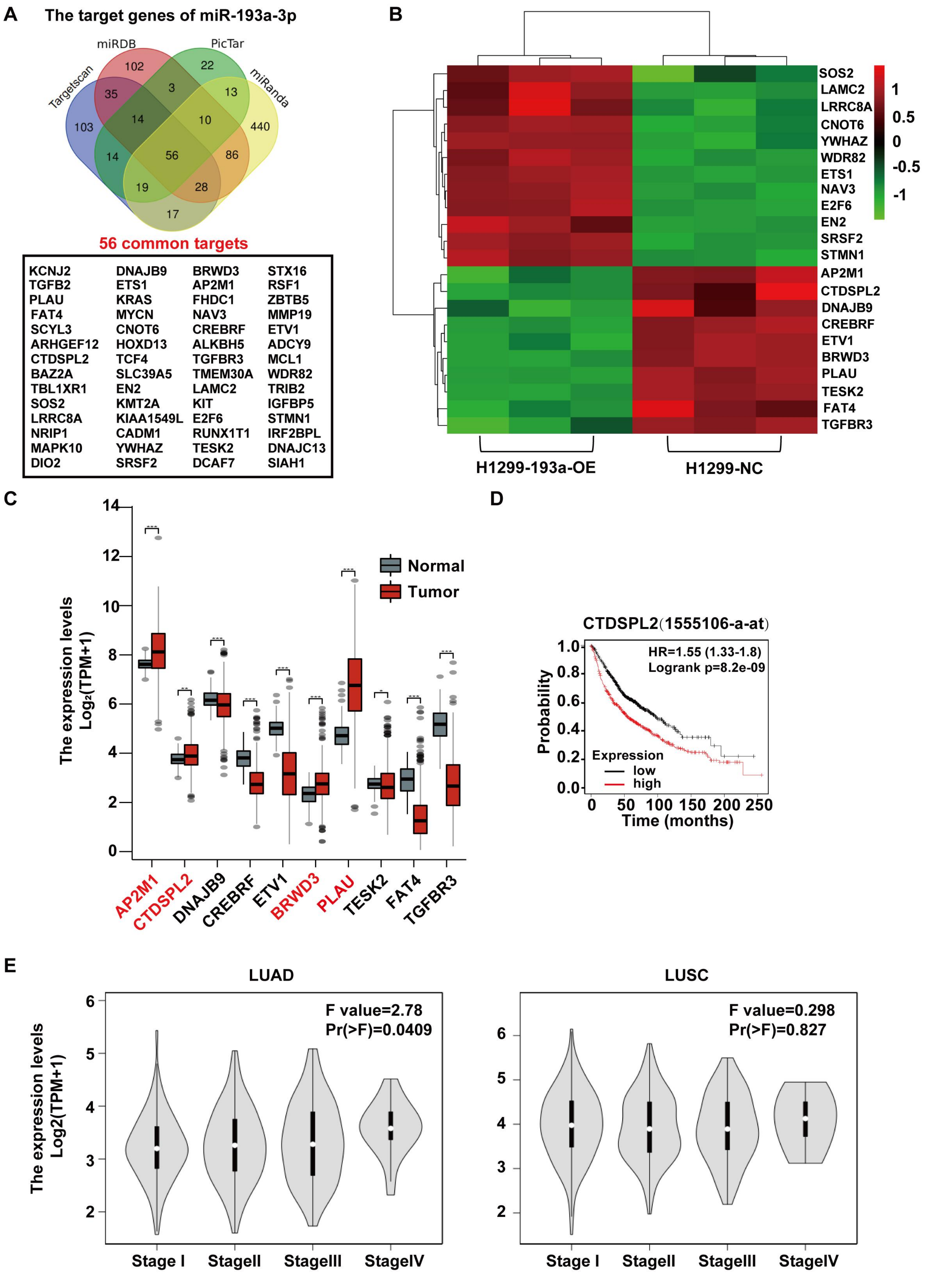

Supplementary Figure 3

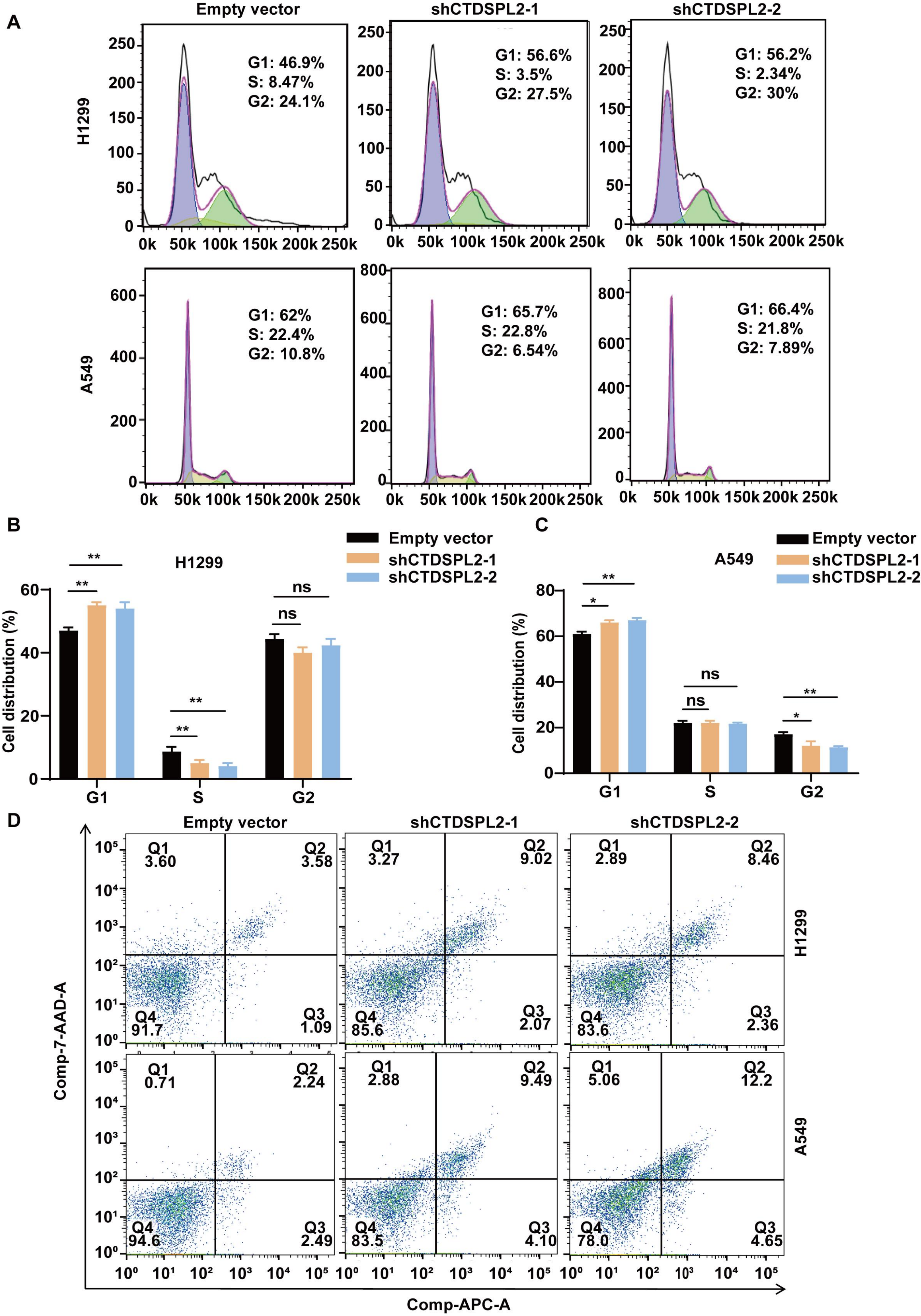

Supplementary Figure 4

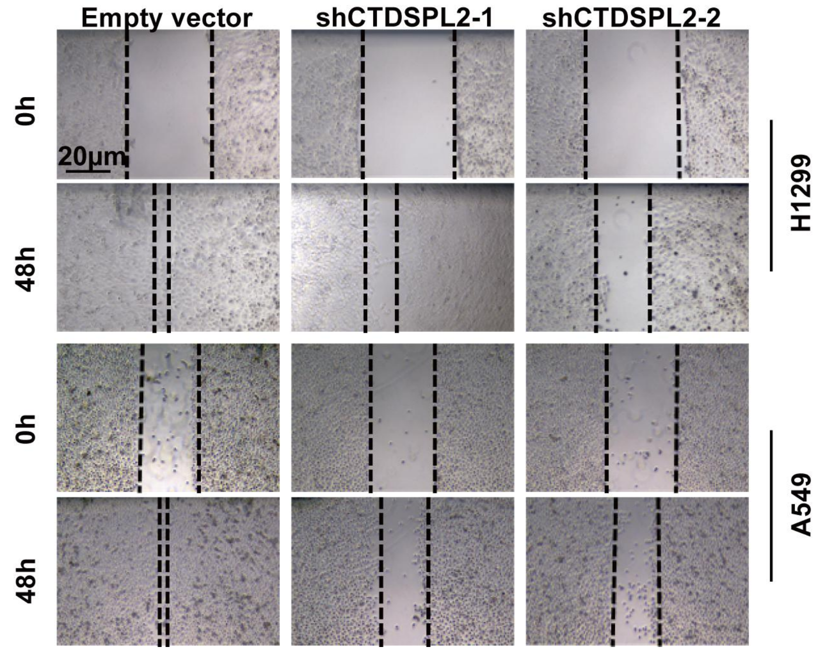

Supplementary Figure 5

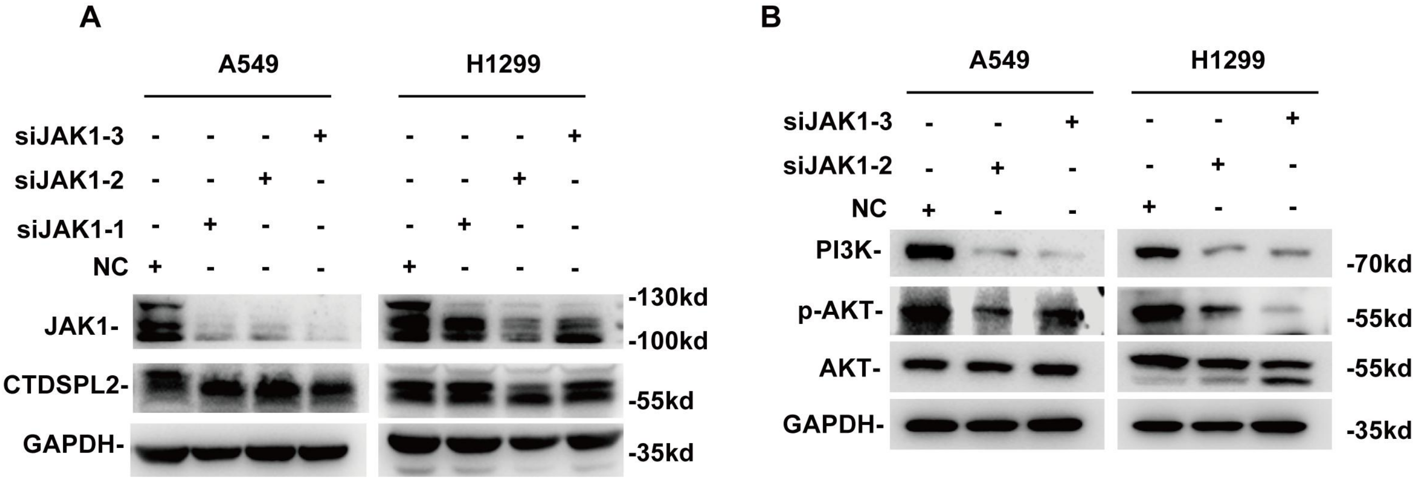

Supplementary Figure 6

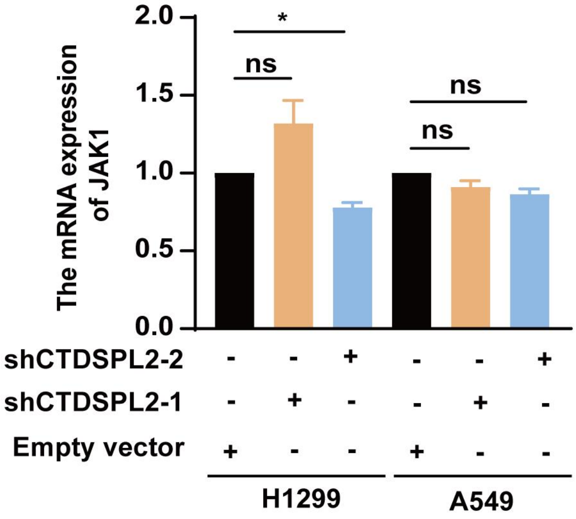

Supplementary Table 1: Clinical information of NSCLC tissues

Variable	Number
**Gender**	
Female	2
Male	12
**Age (years)**	
Mean	64
**Stage**	
Normal lung tissues	14
Ⅰ-Ⅱ	7
Ⅲ-Ⅳ	1
Unknown	6

Supplementary Table 2: Sequences of shRNA and siRNA

**shCTDSPL2-1**	GCACACAGATTTAATGGATAA
**shCTDSPL2-2**	GCTCTCAGTTACAATCAA
**mouse-shCTDSPL2-1**	CCGGGTGTACAAGGAAACTATATAACTCGAGTTATATAGTTTCCTTGTACACTTTTTT
**mouse-shCTDSPL2-2**	CCGGGCTCCCAGTTACAATCAGTTTCTCGAGAAACTGATTGTAACTGGGAGCTTTTTT
**siJAK1-1**	5'-CCACAUAGCUGAUCUGAAATT-3'
	3'-UUUCAGAUCAGCUAUGUGGTT-5'
**siJAK1-2**	5'-CGGGAAGCCUUAAGGAAUATT-3'
	3'-UAUUCCUUAAGGCUUCCCGTT-5'
**siJAK1-3**	5'-CUGAAGAGAAGAAGAUAAATT-3'
	3'-UUUAUCUUCUUCUCUUCAGTT-5'

Supplementary Table 3: Sequences of primers for qRT-PCR

Primer name	Primer sequence
**CTDSPL2**	F: 5'-TGGAACGTCAGGATCAGATTCTC-3'
	R: 5'-GATGGTCTCACTTGAACTGCTTGA-3'
**JAK1**	F: 5'-CTTTGCCCTGTATGACGAGAAC-3'
	R: 5'-ACCTCATCCGGTAGTGGAGC-3'
**GAPDH**	F: 5'-GGAGCGAGATCCCTCCAAAAT-3'
	R: 5'-GGCTGTTGTCATACTTCTCATGG-3'
**miR-193a-RT**	5'-GTCGTATCCAGTGCAGGGTCCGAGGTATTC GCACTGGATACGACACTGGG-3'
**miR-193a**	F: 5'-CGCGAACTGGCCTACAAAGT-3'
	R: 5'-AGTGCAGGGTCCGAGGTATT-3'
**U6-RT**	5'-GCTTCGGCAGCACATATACTAAAAT-3'
**U6**	F: 5'-GCTTCGGCAGCACATATACTAAAAT-3'
	R: 5'-CGCTTCACGAATTTGCGTGTCAT-3'
