# Supplementary material for: CTDSPL2 promotes the progression of non-small lung cancer through PI3K/AKT signaling via JAK1
Source: Cell Death Discov. 2024 Aug 29;10:389. doi: 10.1038/s41420-024-02162-5 (PMC11362329; doi:10.1038/s41420-024-02162-5)

Figure 1G

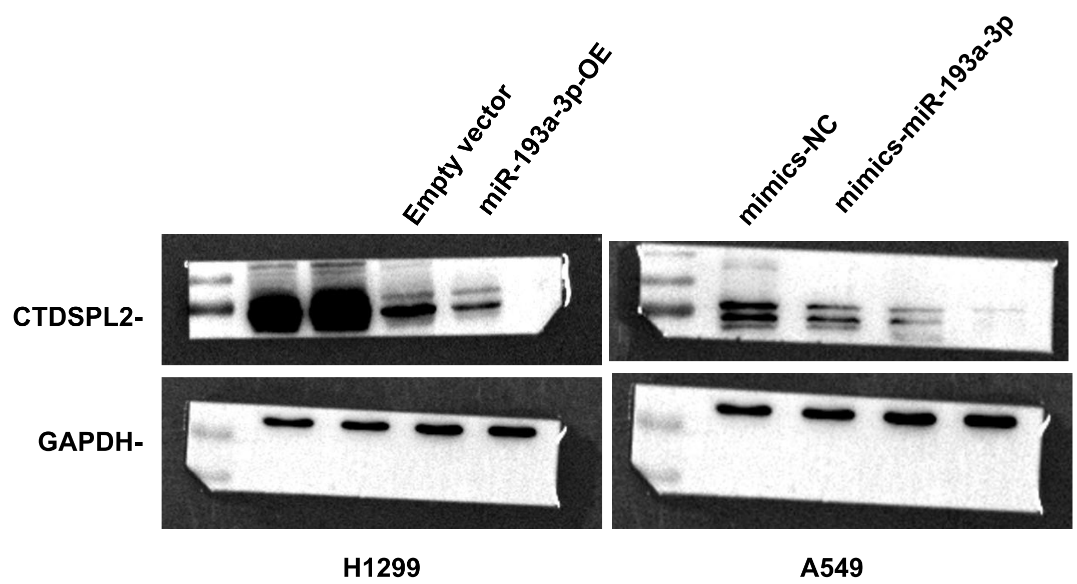

Figure 2B

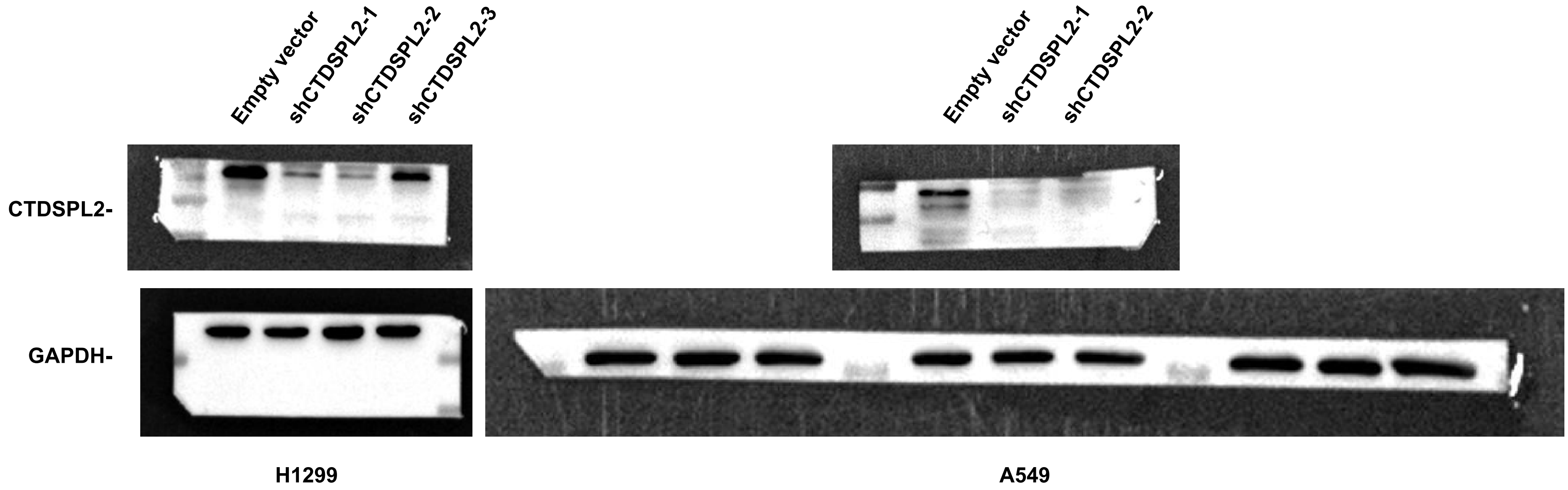

Figure 3A

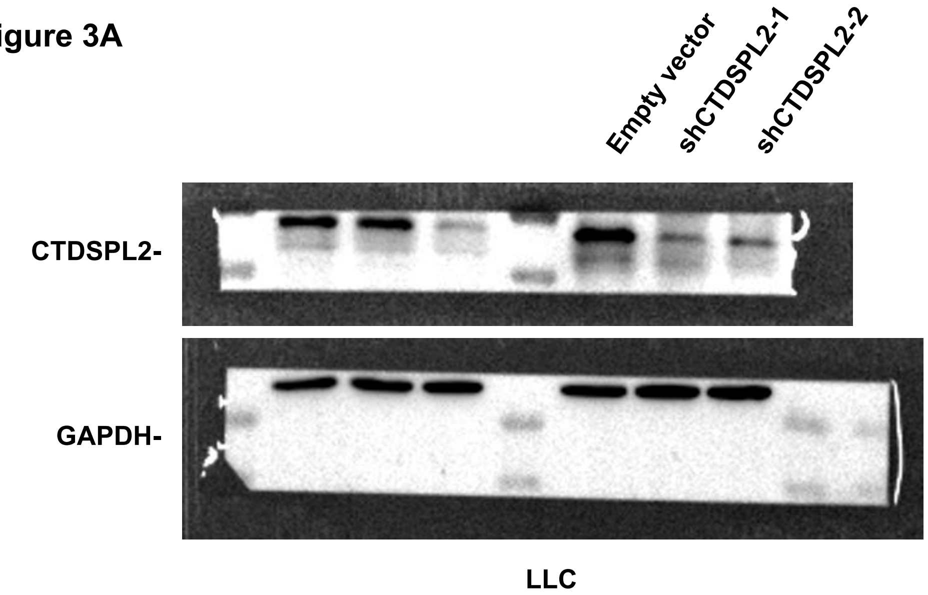

Figure 3E

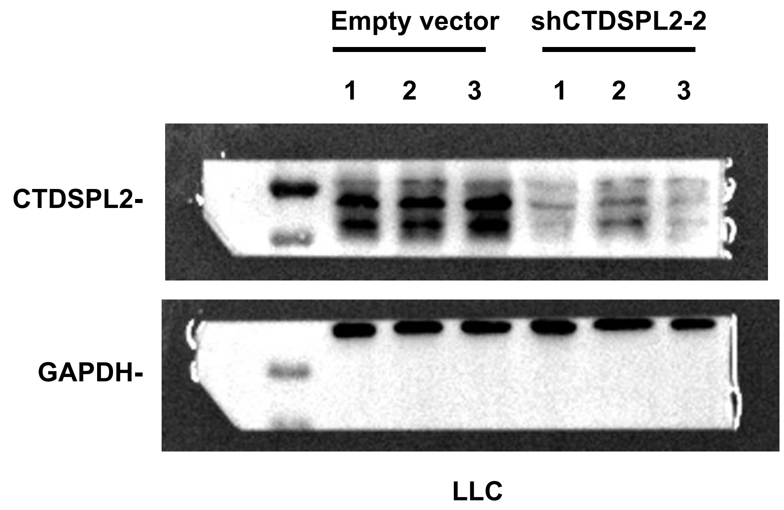

Figure 4B

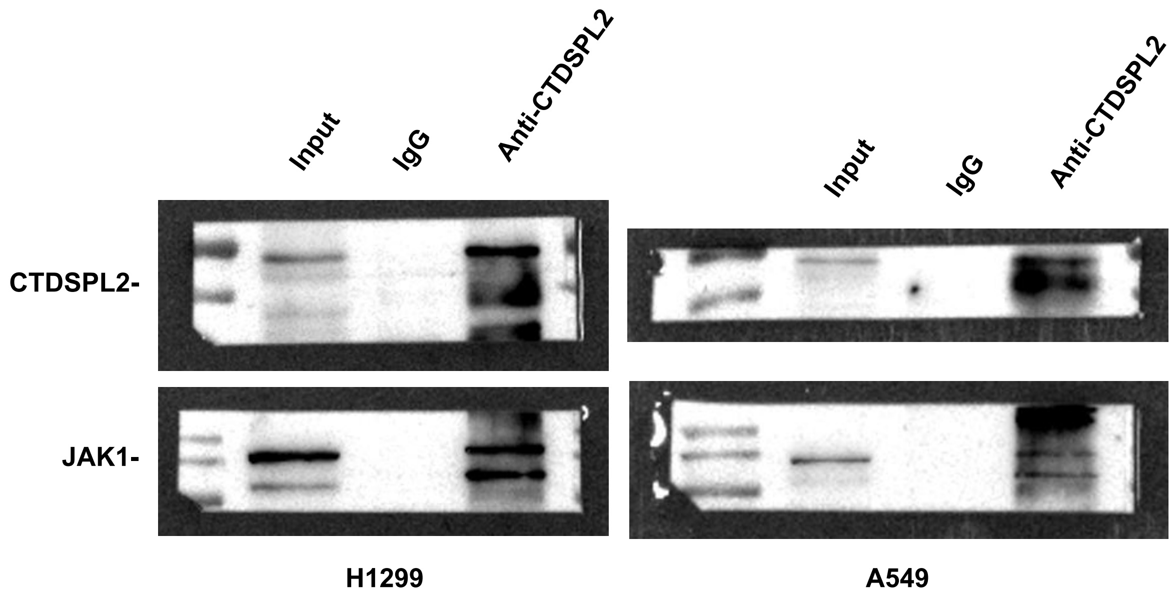

Figure 4C

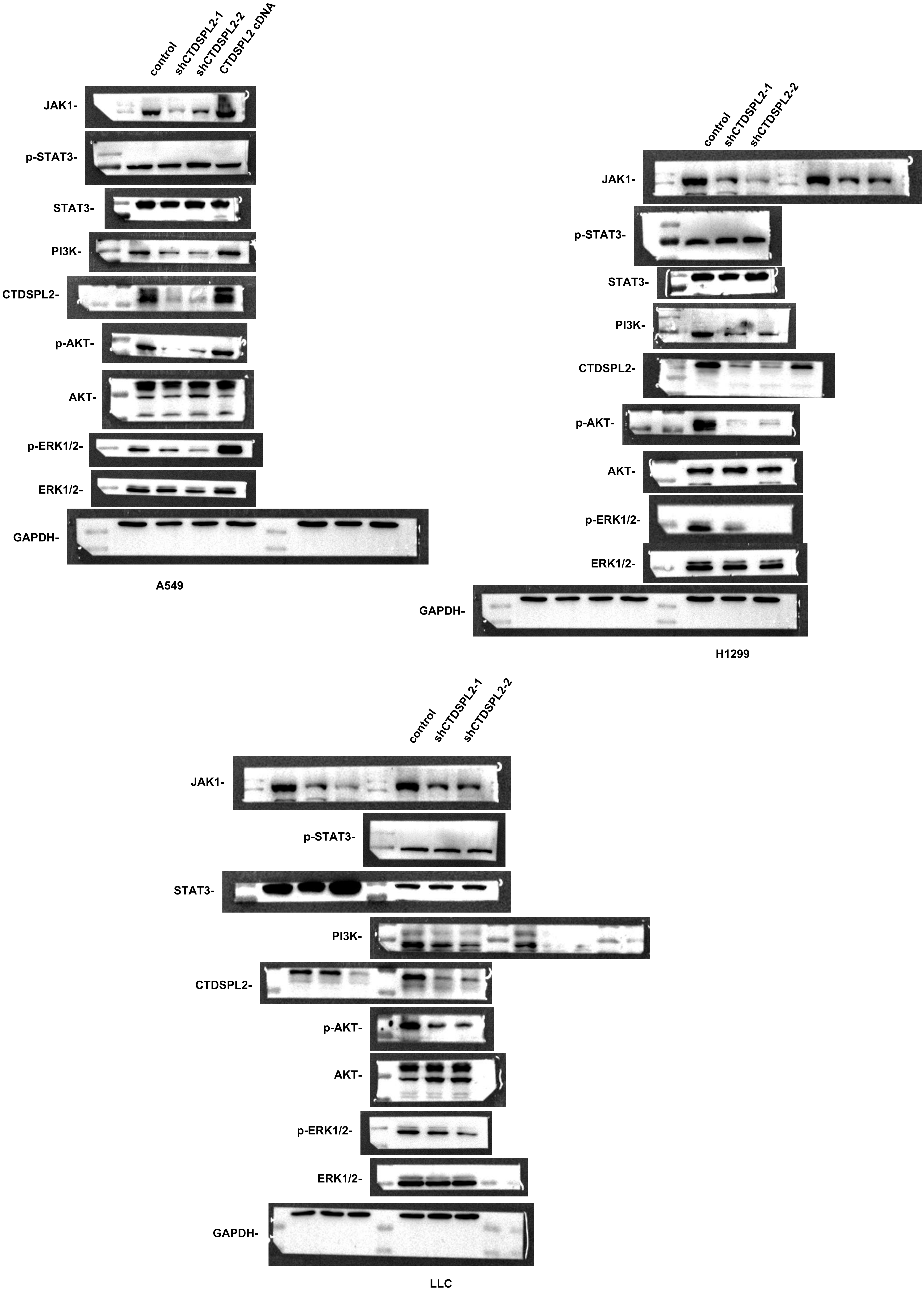
control
shCTDSPL2-1
shCTDSPL2-2
CTDSPL2 cDNA
JAK1-
p-STAT3-
STAT3-
PI3K-
CTDSPL2-
p-AKT-
AKT-
p-ERK1/2-
ERK1/2-
GAPDH-
A549
control
shCTDSPL2-1
shCTDSPL2-2
JAK1-
p-STAT3-
STAT3-
PI3K-
CTDSPL2-
p-AKT-
AKT-
p-ERK1/2-
ERK1/2-
GAPDH-
H1299
control
shCTDSPL2-1
shCTDSPL2-2
JAK1-
p-STAT3-
STAT3-
PI3K-
CTDSPL2-
p-AKT-
AKT-
p-ERK1/2-
ERK1/2-
GAPDH-
LLC

Figure 4D

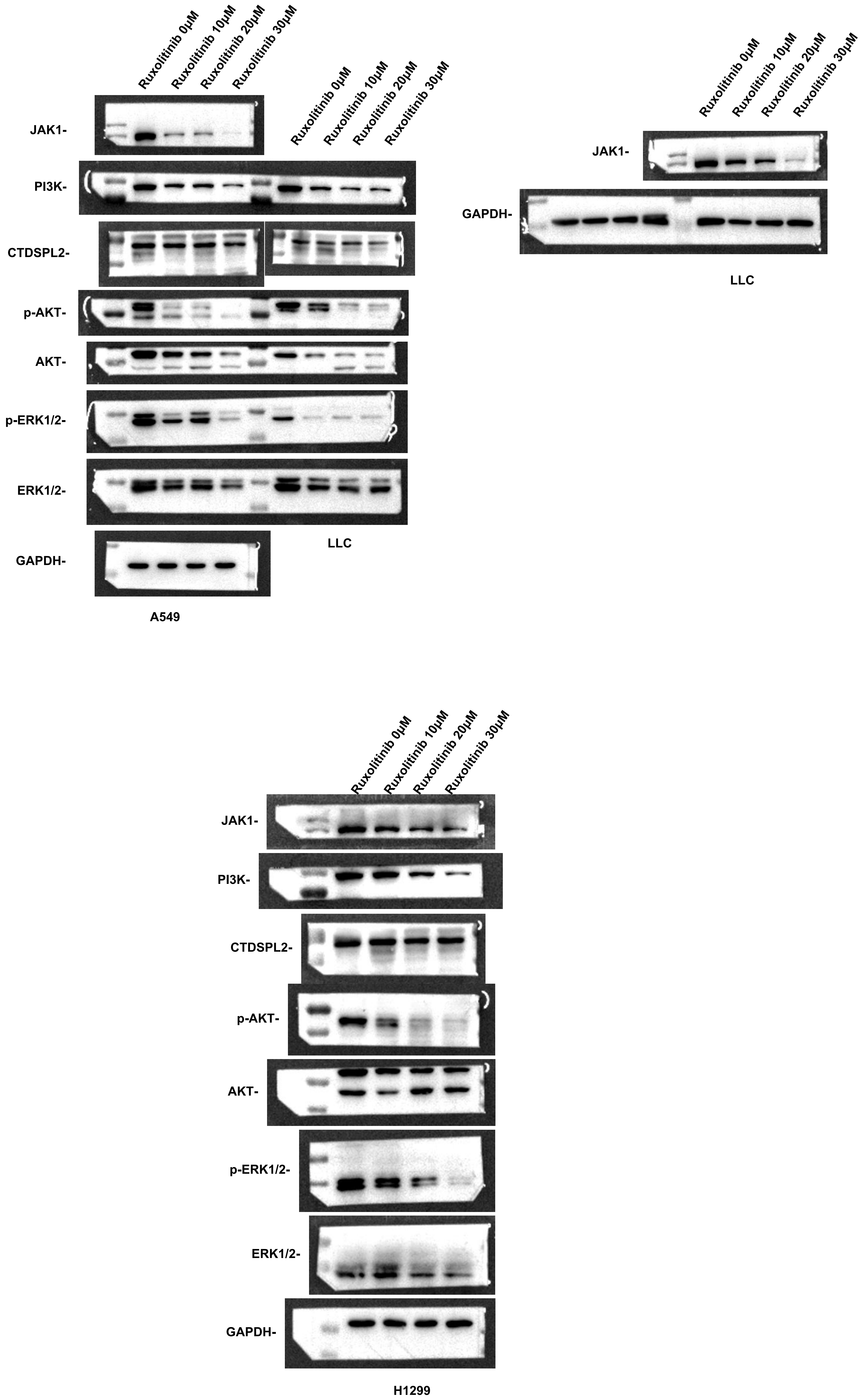
Ruxolitinib 0μM
Ruxolitinib 10μM
Ruxolitinib 20μM
Ruxolitinib 30μM
Ruxolitinib 0μM
Ruxolitinib 10μM
Ruxolitinib 20μM
Ruxolitinib 30μM
JAK1-
PI3K-
CTDSPL2-
p-AKT-
AKT-
p-ERK1/2-
ERK1/2-
GAPDH-
LLC
A549
Ruxolitinib 0μM
Ruxolitinib 10μM
Ruxolitinib 20μM
Ruxolitinib 30μM
JAK1-
GAPDH-
LLC
Ruxolitinib 0μM
Ruxolitinib 10μM
Ruxolitinib 20μM
Ruxolitinib 30μM
JAK1-
PI3K-
CTDSPL2-
p-AKT-
AKT-
p-ERK1/2-
ERK1/2-
GAPDH-
H1299

Supplementary Figure 5A

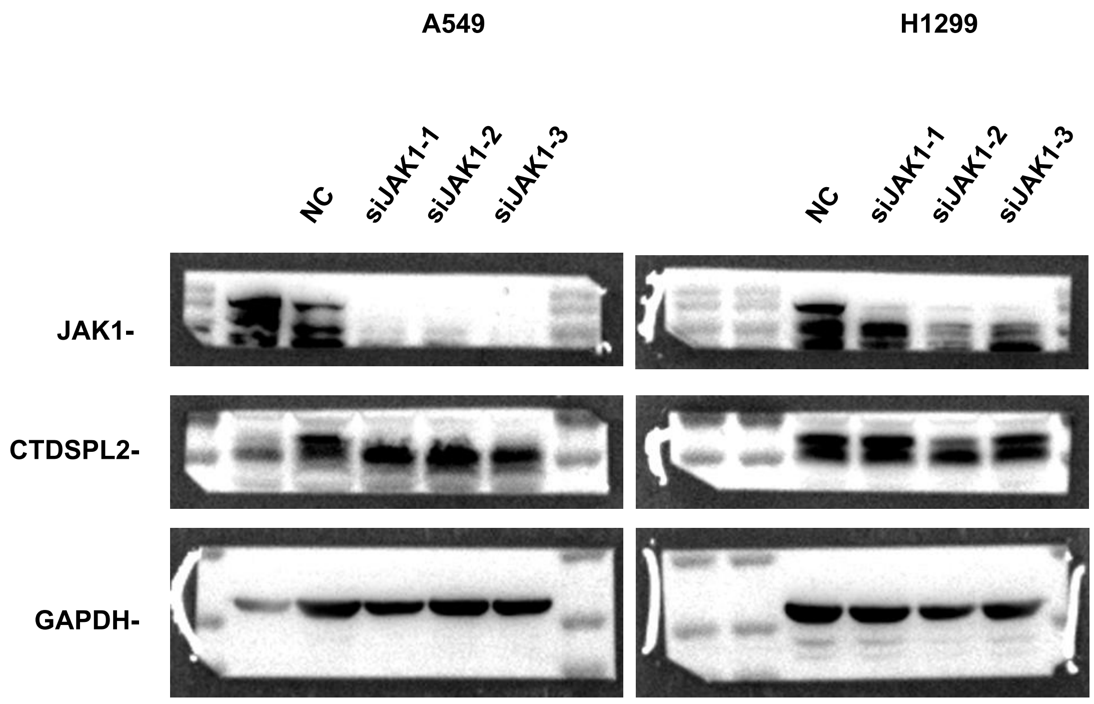

Supplementary Figure 5B

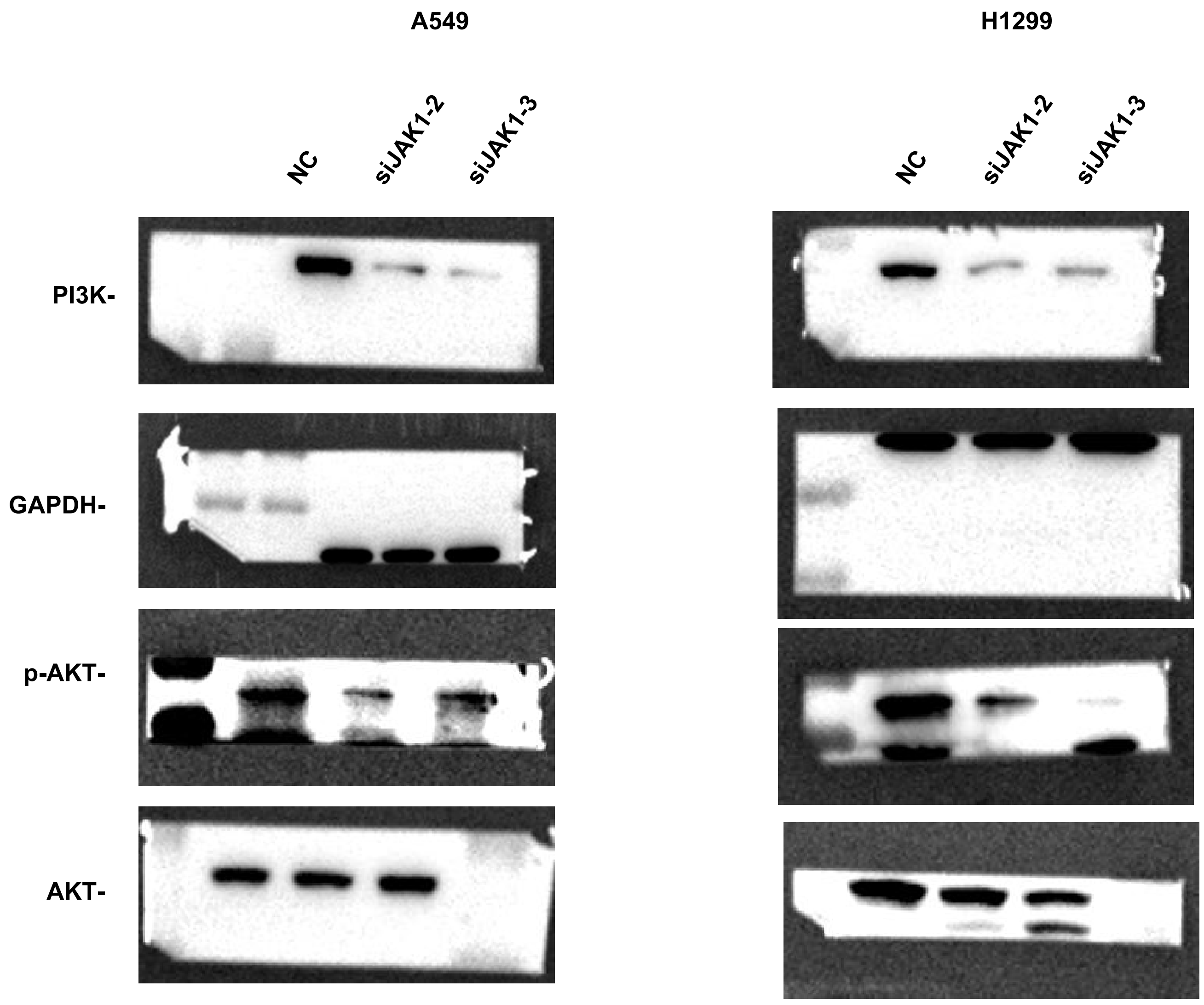

Supplement: Supplementary file 2 — SUPPLEMENTAL MATERIAL-original WB [file 41420_2024_2162_MOESM2_ESM.pdf]
